# Crystal structure of bis­(1,4-di­aza­bicyclo­[2.2.2]octan-1-ium) thio­sulfate dihydrate

**DOI:** 10.1107/S2056989016001535

**Published:** 2016-02-03

**Authors:** Gorgui Awa Seck, Aboubacary Sene, Libasse Diop, Thierry Maris

**Affiliations:** a Laboratoire de Chimie Minérale et Analytique, Département de Chimie, Faculté des Sciences et Techniques, Université Cheikh Anta Diop, Dakar, Sénégal; b Département de Chimie, Université de Montréal, 2900 Boulevard Édouard-Montpetit, Montréal, Québec, H3C 3J7, Canada

**Keywords:** crystal structure, DABCOH^+^ cations, hydrogen bonds, thio­sulfate anion, supra­molecular structure

## Abstract

The crystal structure of the hydrated title salt, 2C_6_H_13_N_2_^+^·S_2_O_3_^2−^·2H_2_O, contains a centrosymmetric cyclic motif of eight hydrogen-bonded mol­ecular subunits. Two DABCOH^+^ cations (DABCO = 1,4-di­aza­bicyclo­[2.2.2]octa­ne) are linked to two water mol­ecules and two thio­sulfate anions *via* O—H⋯N and O—H⋯O hydrogen bonds, respectively. Two other water mol­ecules close the cyclic motif through O—H⋯O contacts to the first two water mol­ecules and to the two thio­sulfate anions. A second pair of DABCOH^+^ cations is N—H⋯O hydrogen bonded to the two anions and is pendant to the ring. Adjacent cyclic motifs are bridged into a block-like arrangement extending along [100] through O—H⋯O inter­actions involving the second pair of water mol­ecules and neighbouring thio­sulfate anions.

## Abstract 2

The crystal structure of the title salt hydrate contains discrete DABCOH^+^ cations (DABCO = 1,4-di­aza­bicyclo­[2.2.2]octa­ne), thio­sulfate anions and lattice water mol­ecules. The three mol­ecular components are held together through hydrogen bonds.

## Chemical context   

The title thio­sulfate was isolated accidentally when thio­acetamide was mixed in ethanol with DABCO (1,4-di­aza­bicyclo­[2.2.2]octa­ne), leading to the formation of the thio­sulfate anion *in situ*.
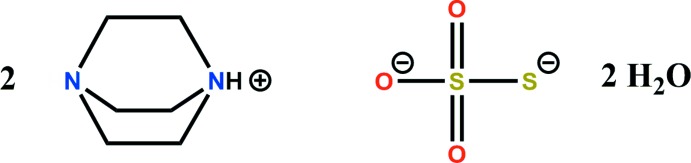


## Structural commentary   

**Figure 1:**
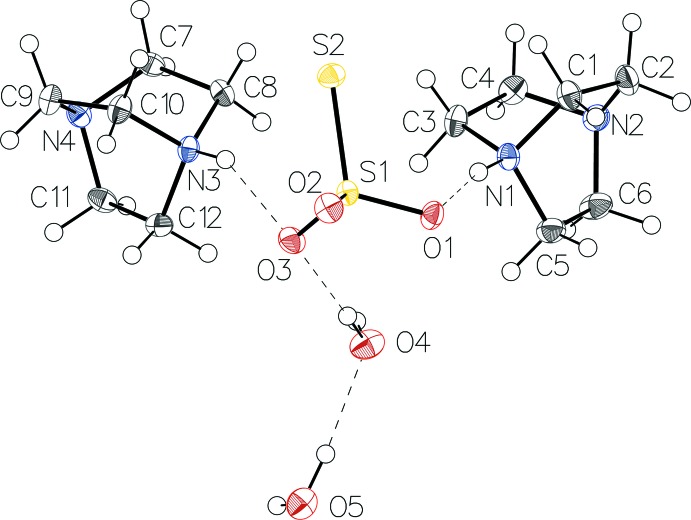
The asymmetric unit of (I), with displacement ellipsoids drawn at the 50% probability level. Hydrogen bonds are shown as dashed lines.

The asymmetric unit (Fig. 1 ▸) consists of one thio­sulfate anion, two monoprotonated DABCOH^+^ cations and two water mol­ecules. The thio­sulfate anion exhibits approximate *C*_3*v*_ symmetry. However, in the crystal it has *C*_1_ symmetry with S—O distances in the range 1.4688 (8) to 1.4898 (8) Å and an S—S bond length of 2.0047 (4) Å, and O—S—O and S—S—O angles ranging from 107.47 (4) to 110.48 (5)°. In both DABCOH^+^ cations, the three N—C bonds involving the protonated N atom are elongated [mean value 1.499 (2) Å] compared to the three N—C bonds involving the non-protonated N atoms [mean value 1.472 (4) Å].

## Supra­molecular features   

**Figure 2:**
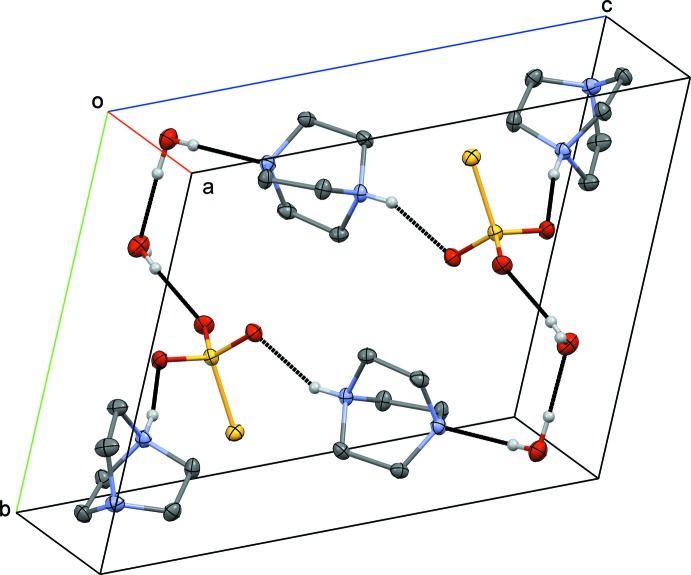
View of the content of one unit cell, showing the hydrogen-bonded macrocycle made up from the asymmetric unit and its inversion-symmetry-related counterpart. H atoms not involved in hydrogen bonding (black dotted lines) are omitted for clarity.

**Figure 3:**
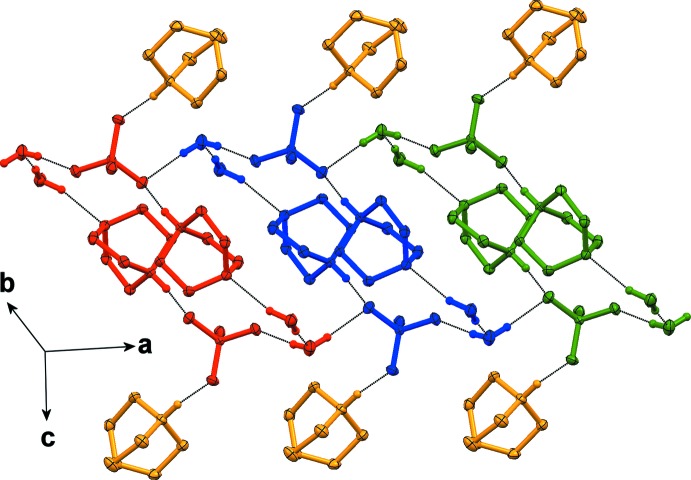
View of three successive hydrogen-bonded cycles displayed in red, blue and green. Pendant DABCOH^+^ cations are shown in orange. H atoms not involved in hydrogen bonding (black dotted lines) are omitted for clarity.

The thio­sulfate anion is linked *via* charge-assisted N—H⋯O hydrogen bonds to two DABCOH^+^ cations. The third oxygen atom (O2) of the anion acts as a hydrogen-bond acceptor for one of the water mol­ecules (O4). The second hydrogen bond involving this water mol­ecule is directed towards a symmetry-related thio­sulfate anion. The second water mol­ecule (O5) is the donor of one O—H⋯O hydrogen bond to the other water mol­ecule and of one N—H⋯O hydrogen bond to one of the DABCOH^+^ cations. Numerical details of the hydrogen-bonding inter­actions are given in Table 1 ▸. This arrangement leads to the formation of a centrosymmetric cyclic motif consisting of eight hydrogen-bonded mol­ecules with two pendant DABCOH^+^ cations (Fig. 2 ▸). Adjacent cyclic motifs are bridged through O4—H44⋯O3 contacts into supra­molecular blocks running along [100] (Fig. 3 ▸).

## Database survey   

A search in the Cambridge Structural Database (CSD Version 5.36 with three updates; Groom & Allen, 2014 ▸) for salts with isolated thio­sulfate anions returned 25 records with ten of them featuring a metal complex for the cationic part. Entries with simple protonated amine functionalities include structures with *tert*-butyl­ammonium (Okuniewski *et al.*, 2013 ▸) and its hydrate (Dabrowska & Chojnacki, 2014 ▸), cyclo­hexyl­ammonium (Dabrowska & Chojnacki, 2014 ▸), tetra­methyl­ammonium tetra­hydrate (Yang & Ng, 2011 ▸), tetra­ethyl­ammonium dihydrate (Leyten *et al.*, 1988 ▸), iso­propyl­ammonium (Okuniewski *et al.*, 2013 ▸), piperazinium (Srinivasan *et al.*, 2011 ▸) and adamantanaminium (Jiang *et al.*, 1998 ▸). The thio­sulfate anion has also been encapsulated in protonated aza­cryptands ligands (Maubert *et al.*, 2001 ▸; Nelson *et al.*, 2004 ▸).

## Synthesis and crystallization   

Crystals suitable for a single-crystal X-ray diffraction study were isolated from a clear ethano­lic solution of thio­acetamide and DABCO in an equimolar ratio.

## Refinement   

Crystal data, data collection and structure refinement details are summarized in Table 2 ▸. All H atoms were located from Fourier difference maps and freely refined.

## Supplementary Material

Crystal structure: contains datablock(s) global, I. DOI: 10.1107/S2056989016001535/wm5264sup1.cif

Structure factors: contains datablock(s) I. DOI: 10.1107/S2056989016001535/wm5264Isup2.hkl

Supporting information file. DOI: 10.1107/S2056989016001535/wm5264Isup3.cml

CCDC reference: 1449673

Additional supporting information:  crystallographic information; 3D view; checkCIF report

## Figures and Tables

**Table 1:** Hydrogen-bond geometry (Å, °)

*D*—H⋯*A*	*D*—H	H⋯*A*	*D*⋯*A*	*D*—H⋯*A*
N1—H1⋯O1	0.887 (17)	1.861 (17)	2.7380 (12)	169.6 (15)
N3—H3⋯O3	0.850 (18)	2.030 (17)	2.8003 (12)	150.4 (15)
O4—H4*C*⋯O2^i^	0.79 (2)	2.02 (2)	2.8121 (13)	173.4 (18)
O4—H4*D*⋯O3	0.81 (2)	2.04 (2)	2.8511 (13)	175.1 (18)
O5—H5*C*⋯N4^ii^	0.85 (2)	2.10 (2)	2.9273 (13)	163.9 (19)
O5—H5*D*⋯O4	0.91 (2)	1.94 (2)	2.8449 (14)	173.2 (19)

Symmetry codes: (i) 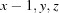; (ii) 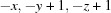.

**Table 2:** Experimental details

Crystal data	
Chemical formula	2C_6_H_13_N_2_^+^·S_2_O_3_^2−^·2H_2_O
*M*_r_	374.52
Crystal system, space group	Triclinic, *P* 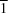
Temperature (K)	100
*a*, *b*, *c* (Å)	6.5063 (2), 10.5966 (3), 13.2066 (4)
α, β, γ (°)	105.951 (1), 92.065 (1), 96.550 (1)
*V* (Å^3^)	867.59 (5)
*Z*	2
Radiation type	Ga *K*α, λ = 1.34139 Å
μ (mm^−1^)	1.98
Crystal size (mm)	0.51 × 0.18 × 0.06

Data collection	
Diffractometer	Bruker Venture Metaljet
Absorption correction	Multi-scan (*SADABS*; Krause *et al.*, 2015 ▸)
*T*_min_, *T*_max_	0.170, 0.311
No. of measured, independent and observed [*I* > 2σ(*I*)] reflections	24479, 3965, 3865
*R*_int_	0.040
(sin θ/λ)_max_ (Å^−1^)	0.650

Refinement	
*R*[*F* ^2^ > 2σ(*F* ^2^)], *wR*(*F* ^2^), *S*	0.029, 0.079, 1.07
No. of reflections	3965
No. of parameters	328
H-atom treatment	All H-atom parameters refined
Δρ_max_, Δρ_min_ (e Å^−3^)	0.45, −0.31

Computer programs: *APEX2* and *SAINT* (Bruker, 2014 ▸), *SHELXT* (Sheldrick, 2015*a* ▸), *SHELXL2014* (Sheldrick, 2015*b* ▸), *OLEX2* (Dolomanov *et al.*, 2009 ▸), *Mercury* (Macrae *et al.*, 2008 ▸) and *publCIF* (Westrip, 2010 ▸).

## supplementary crystallographic information

### Crystal data

**Table:**

2C_6_H_13_N_2_^+^·S_2_O_3_^2^^−^·2H_2_O	*Z* = 2
*M**_r_* = 374.52	*F*(000) = 404
Triclinic, *P*1	*D*_x_ = 1.434 Mg m^−^^3^
*a* = 6.5063 (2) Å	Ga *K*α radiation, λ = 1.34139 Å
*b* = 10.5966 (3) Å	Cell parameters from 9855 reflections
*c* = 13.2066 (4) Å	θ = 3.0–60.7°
α = 105.951 (1)°	µ = 1.98 mm^−^^1^
β = 92.065 (1)°	*T* = 100 K
γ = 96.550 (1)°	Platelet, clear light colourless
*V* = 867.59 (5) Å^3^	0.51 × 0.18 × 0.06 mm

### Data collection

**Table:**

Bruker Venture Metaljet diffractometer	3965 independent reflections
Radiation source: Metal Jet, Gallium Liquid Metal Jet Source	3865 reflections with *I* > 2σ(*I*)
Helios MX Mirror Optics monochromator	*R*_int_ = 0.040
Detector resolution: 10.24 pixels mm^-1^	θ_max_ = 60.6°, θ_min_ = 3.0°
ω and φ scans	*h* = −8→8
Absorption correction: multi-scan (*SADABS*; Krause *et al.*, 2015)	*k* = −13→13
*T*_min_ = 0.170, *T*_max_ = 0.311	*l* = −17→17
24479 measured reflections	

### Refinement

**Table:**

Refinement on *F*^2^	Primary atom site location: structure-invariant direct methods
Least-squares matrix: full	Hydrogen site location: difference Fourier map
*R*[*F*^2^ > 2σ(*F*^2^)] = 0.029	All H-atom parameters refined
*wR*(*F*^2^) = 0.079	*w* = 1/[σ^2^(*F*_o_^2^) + (0.0453*P*)^2^ + 0.2876*P*] where *P* = (*F*_o_^2^ + 2*F*_c_^2^)/3
*S* = 1.07	(Δ/σ)_max_ = 0.001
3965 reflections	Δρ_max_ = 0.45 e Å^−^^3^
328 parameters	Δρ_min_ = −0.31 e Å^−^^3^
0 restraints	

### Special details

**Table:**

Experimental. X-ray crystallographic data for I were collected from a single-crystal sample, which was mounted on a loop fiber. Data were collected using a Bruker Venture diffractometer equipped with a Photon 100 CMOS Detector, a Helios MX optics and a Kappa goniometer. The crystal-to-detector distance was 4.0 cm, and the data collection was carried out in 1024 *x* 1024 pixel mode.
Geometry. All e.s.d.'s (except the e.s.d. in the dihedral angle between two l.s. planes) are estimated using the full covariance matrix. The cell e.s.d.'s are taken into account individually in the estimation of e.s.d.'s in distances, angles and torsion angles; correlations between e.s.d.'s in cell parameters are only used when they are defined by crystal symmetry. An approximate (isotropic) treatment of cell e.s.d.'s is used for estimating e.s.d.'s involving l.s. planes.

### Fractional atomic coordinates and isotropic or equivalent isotropic displacement parameters (Å^2^)

**Table:**

	*x*	*y*	*z*	*U*_iso_*/*U*_eq_	
N1	0.37120 (14)	0.25575 (9)	0.89793 (7)	0.01709 (19)	
H1	0.474 (3)	0.2918 (16)	0.8688 (13)	0.027 (4)*	
N2	0.07772 (15)	0.16056 (10)	0.98667 (8)	0.0204 (2)	
C1	0.44755 (18)	0.15612 (12)	0.94669 (9)	0.0204 (2)	
H1A	0.477 (2)	0.0796 (16)	0.8860 (13)	0.026 (4)*	
H1B	0.572 (3)	0.1986 (16)	0.9896 (13)	0.026 (4)*	
C2	0.27163 (19)	0.11153 (12)	1.00935 (9)	0.0227 (2)	
H2A	0.308 (3)	0.1446 (18)	1.0861 (15)	0.036 (4)*	
H2B	0.250 (3)	0.0161 (17)	0.9902 (13)	0.029 (4)*	
C3	0.19715 (18)	0.18911 (13)	0.81671 (9)	0.0211 (2)	
H3A	0.254 (2)	0.1287 (16)	0.7618 (13)	0.025 (4)*	
H3B	0.149 (3)	0.2574 (17)	0.7886 (13)	0.032 (4)*	
C4	0.03175 (18)	0.12017 (12)	0.87190 (9)	0.0215 (2)	
H4A	−0.102 (3)	0.1438 (16)	0.8578 (13)	0.028 (4)*	
H4B	0.027 (2)	0.0273 (17)	0.8492 (13)	0.028 (4)*	
C5	0.29855 (19)	0.36527 (12)	0.98198 (10)	0.0226 (2)	
H5A	0.268 (3)	0.4332 (17)	0.9503 (13)	0.029 (4)*	
H5B	0.417 (3)	0.3988 (17)	1.0355 (13)	0.030 (4)*	
C6	0.1044 (2)	0.30577 (12)	1.02479 (11)	0.0263 (3)	
H6A	0.118 (3)	0.3316 (18)	1.1015 (15)	0.037 (4)*	
H6B	−0.020 (3)	0.3362 (17)	0.9978 (13)	0.032 (4)*	
N3	0.32970 (14)	0.27267 (9)	0.50138 (7)	0.01556 (18)	
H3	0.423 (3)	0.2931 (16)	0.5520 (13)	0.028 (4)*	
N4	0.05427 (14)	0.20524 (9)	0.34935 (7)	0.01749 (19)	
C7	0.02619 (17)	0.11478 (11)	0.41613 (9)	0.0180 (2)	
H7A	0.050 (2)	0.0289 (16)	0.3785 (12)	0.022 (4)*	
H7B	−0.117 (2)	0.1056 (15)	0.4338 (11)	0.020 (3)*	
C8	0.17326 (17)	0.16570 (11)	0.51711 (9)	0.0182 (2)	
H8A	0.107 (2)	0.2033 (15)	0.5776 (12)	0.020 (3)*	
H8B	0.248 (2)	0.1029 (16)	0.5297 (12)	0.024 (4)*	
C9	0.26331 (18)	0.20134 (12)	0.30931 (9)	0.0197 (2)	
H9A	0.265 (2)	0.1127 (16)	0.2591 (12)	0.024 (4)*	
H9B	0.287 (2)	0.2726 (16)	0.2771 (13)	0.026 (4)*	
C10	0.43012 (17)	0.22308 (12)	0.40030 (8)	0.0185 (2)	
H10A	0.482 (2)	0.1435 (16)	0.4030 (12)	0.024 (4)*	
H10B	0.540 (2)	0.2897 (15)	0.3999 (12)	0.022 (4)*	
C11	0.03966 (18)	0.34059 (11)	0.41524 (9)	0.0202 (2)	
H11A	−0.091 (2)	0.3368 (14)	0.4464 (11)	0.019 (3)*	
H11B	0.039 (2)	0.3977 (16)	0.3696 (13)	0.026 (4)*	
C12	0.22526 (17)	0.39014 (11)	0.49767 (9)	0.0188 (2)	
H12A	0.328 (3)	0.4497 (16)	0.4780 (13)	0.027 (4)*	
H12B	0.184 (2)	0.4274 (15)	0.5693 (12)	0.023 (4)*	
S1	0.73999 (4)	0.36034 (2)	0.71916 (2)	0.01430 (8)	
S2	0.66847 (4)	0.16633 (3)	0.64755 (2)	0.01940 (9)	
O1	0.70661 (12)	0.38533 (8)	0.83256 (6)	0.01974 (17)	
O2	0.95658 (12)	0.40399 (8)	0.70450 (6)	0.02108 (18)	
O3	0.59842 (12)	0.43146 (8)	0.67012 (6)	0.02058 (17)	
O4	0.31661 (15)	0.56983 (9)	0.80581 (8)	0.02666 (19)	
H4C	0.211 (3)	0.5246 (19)	0.7817 (15)	0.035 (5)*	
H4D	0.402 (3)	0.5326 (18)	0.7700 (15)	0.036 (5)*	
O5	0.31448 (14)	0.83414 (9)	0.79500 (7)	0.02522 (19)	
H5C	0.196 (3)	0.830 (2)	0.7642 (16)	0.048 (5)*	
H5D	0.308 (3)	0.751 (2)	0.8025 (16)	0.049 (5)*	

### Atomic displacement parameters (Å^2^)

**Table:**

	*U*^11^	*U*^22^	*U*^33^	*U*^12^	*U*^13^	*U*^23^
N1	0.0159 (4)	0.0205 (4)	0.0158 (4)	0.0012 (3)	0.0031 (3)	0.0069 (3)
N2	0.0205 (5)	0.0199 (5)	0.0201 (5)	0.0000 (4)	0.0066 (4)	0.0045 (4)
C1	0.0186 (5)	0.0246 (6)	0.0204 (5)	0.0038 (4)	0.0005 (4)	0.0097 (4)
C2	0.0251 (6)	0.0253 (6)	0.0199 (5)	−0.0001 (5)	0.0018 (4)	0.0115 (4)
C3	0.0180 (5)	0.0295 (6)	0.0151 (5)	0.0022 (4)	−0.0008 (4)	0.0059 (4)
C4	0.0161 (5)	0.0236 (6)	0.0226 (5)	−0.0003 (4)	−0.0005 (4)	0.0042 (4)
C5	0.0256 (6)	0.0174 (5)	0.0236 (5)	0.0005 (4)	0.0064 (5)	0.0040 (4)
C6	0.0272 (6)	0.0209 (6)	0.0284 (6)	0.0019 (5)	0.0132 (5)	0.0020 (5)
N3	0.0135 (4)	0.0200 (4)	0.0134 (4)	0.0025 (3)	0.0005 (3)	0.0049 (3)
N4	0.0154 (4)	0.0190 (4)	0.0191 (4)	0.0021 (3)	−0.0008 (3)	0.0072 (3)
C7	0.0156 (5)	0.0160 (5)	0.0230 (5)	0.0014 (4)	0.0014 (4)	0.0069 (4)
C8	0.0175 (5)	0.0201 (5)	0.0201 (5)	0.0033 (4)	0.0031 (4)	0.0104 (4)
C9	0.0178 (5)	0.0263 (6)	0.0143 (5)	0.0013 (4)	0.0009 (4)	0.0052 (4)
C10	0.0138 (5)	0.0256 (5)	0.0154 (5)	0.0041 (4)	0.0030 (4)	0.0039 (4)
C11	0.0178 (5)	0.0181 (5)	0.0269 (6)	0.0047 (4)	0.0001 (4)	0.0091 (4)
C12	0.0194 (5)	0.0156 (5)	0.0211 (5)	0.0029 (4)	0.0029 (4)	0.0043 (4)
S1	0.01214 (13)	0.01792 (14)	0.01419 (13)	0.00319 (9)	0.00261 (9)	0.00605 (9)
S2	0.01877 (14)	0.01794 (14)	0.02064 (14)	0.00461 (10)	0.00074 (10)	0.00317 (10)
O1	0.0211 (4)	0.0228 (4)	0.0144 (4)	0.0000 (3)	0.0044 (3)	0.0044 (3)
O2	0.0139 (4)	0.0256 (4)	0.0247 (4)	0.0016 (3)	0.0051 (3)	0.0085 (3)
O3	0.0199 (4)	0.0209 (4)	0.0228 (4)	0.0063 (3)	−0.0010 (3)	0.0081 (3)
O4	0.0197 (4)	0.0269 (5)	0.0302 (5)	0.0071 (4)	−0.0005 (4)	0.0015 (4)
O5	0.0196 (4)	0.0290 (5)	0.0251 (4)	0.0034 (3)	−0.0016 (3)	0.0047 (3)

### Geometric parameters (Å, º)

**Table:**

N1—H1	0.887 (17)	N4—C7	1.4720 (14)
N1—C1	1.4988 (14)	N4—C9	1.4778 (14)
N1—C3	1.4984 (14)	N4—C11	1.4746 (14)
N1—C5	1.5025 (14)	C7—H7A	0.942 (16)
N2—C2	1.4694 (16)	C7—H7B	0.967 (15)
N2—C4	1.4668 (15)	C7—C8	1.5428 (15)
N2—C6	1.4691 (15)	C8—H8A	0.933 (15)
C1—H1A	1.013 (16)	C8—H8B	0.916 (16)
C1—H1B	0.959 (17)	C9—H9A	0.991 (16)
C1—C2	1.5421 (16)	C9—H9B	0.963 (17)
C2—H2A	0.988 (18)	C9—C10	1.5401 (15)
C2—H2B	0.964 (17)	C10—H10A	0.952 (16)
C3—H3A	0.942 (16)	C10—H10B	0.947 (16)
C3—H3B	0.975 (18)	C11—H11A	0.960 (15)
C3—C4	1.5469 (16)	C11—H11B	0.964 (17)
C4—H4A	0.960 (17)	C11—C12	1.5380 (16)
C4—H4B	0.943 (17)	C12—H12A	0.958 (17)
C5—H5A	0.962 (17)	C12—H12B	0.979 (16)
C5—H5B	0.992 (17)	S1—S2	2.0047 (4)
C5—C6	1.5424 (16)	S1—O1	1.4761 (8)
C6—H6A	0.972 (18)	S1—O2	1.4688 (8)
C6—H6B	0.993 (17)	S1—O3	1.4898 (8)
N3—H3	0.850 (18)	O4—H4C	0.79 (2)
N3—C8	1.4985 (14)	O4—H4D	0.81 (2)
N3—C10	1.4969 (13)	O5—H5C	0.85 (2)
N3—C12	1.4958 (14)	O5—H5D	0.91 (2)
			
C1—N1—H1	109.6 (10)	C12—N3—C8	109.71 (8)
C1—N1—C5	109.73 (9)	C12—N3—C10	109.52 (8)
C3—N1—H1	110.5 (11)	C7—N4—C9	108.87 (9)
C3—N1—C1	109.37 (9)	C7—N4—C11	108.26 (9)
C3—N1—C5	110.23 (9)	C11—N4—C9	108.68 (9)
C5—N1—H1	107.4 (11)	N4—C7—H7A	110.5 (9)
C4—N2—C2	108.23 (9)	N4—C7—H7B	109.8 (9)
C4—N2—C6	108.88 (10)	N4—C7—C8	110.60 (9)
C6—N2—C2	109.51 (10)	H7A—C7—H7B	105.1 (13)
N1—C1—H1A	106.3 (9)	C8—C7—H7A	110.1 (9)
N1—C1—H1B	106.9 (10)	C8—C7—H7B	110.6 (9)
N1—C1—C2	107.31 (9)	N3—C8—C7	107.51 (8)
H1A—C1—H1B	111.4 (13)	N3—C8—H8A	106.7 (9)
C2—C1—H1A	110.7 (9)	N3—C8—H8B	105.6 (10)
C2—C1—H1B	113.7 (10)	C7—C8—H8A	113.8 (9)
N2—C2—C1	111.18 (9)	C7—C8—H8B	113.4 (10)
N2—C2—H2A	108.2 (10)	H8A—C8—H8B	109.2 (13)
N2—C2—H2B	109.3 (10)	N4—C9—H9A	107.4 (9)
C1—C2—H2A	111.0 (11)	N4—C9—H9B	106.9 (9)
C1—C2—H2B	108.9 (10)	N4—C9—C10	110.83 (9)
H2A—C2—H2B	108.2 (14)	H9A—C9—H9B	113.4 (13)
N1—C3—H3A	107.1 (10)	C10—C9—H9A	109.4 (9)
N1—C3—H3B	106.8 (10)	C10—C9—H9B	108.9 (10)
N1—C3—C4	107.21 (9)	N3—C10—C9	107.32 (9)
H3A—C3—H3B	108.1 (14)	N3—C10—H10A	106.7 (9)
C4—C3—H3A	112.5 (10)	N3—C10—H10B	105.8 (9)
C4—C3—H3B	114.6 (10)	C9—C10—H10A	112.9 (9)
N2—C4—C3	111.11 (9)	C9—C10—H10B	112.7 (9)
N2—C4—H4A	107.9 (10)	H10A—C10—H10B	111.0 (13)
N2—C4—H4B	106.8 (10)	N4—C11—H11A	106.2 (9)
C3—C4—H4A	110.0 (10)	N4—C11—H11B	108.1 (10)
C3—C4—H4B	111.5 (10)	N4—C11—C12	110.79 (9)
H4A—C4—H4B	109.5 (14)	H11A—C11—H11B	109.1 (13)
N1—C5—H5A	108.0 (10)	C12—C11—H11A	113.0 (9)
N1—C5—H5B	105.4 (10)	C12—C11—H11B	109.4 (10)
N1—C5—C6	107.54 (9)	N3—C12—C11	107.39 (9)
H5A—C5—H5B	110.3 (14)	N3—C12—H12A	105.8 (10)
C6—C5—H5A	111.7 (10)	N3—C12—H12B	105.9 (9)
C6—C5—H5B	113.5 (10)	C11—C12—H12A	112.4 (10)
N2—C6—C5	110.75 (9)	C11—C12—H12B	113.0 (9)
N2—C6—H6A	108.4 (11)	H12A—C12—H12B	111.7 (13)
N2—C6—H6B	107.4 (10)	O1—S1—S2	109.01 (3)
C5—C6—H6A	109.9 (11)	O1—S1—O3	109.73 (5)
C5—C6—H6B	108.8 (10)	O2—S1—S2	110.12 (4)
H6A—C6—H6B	111.6 (14)	O2—S1—O1	110.48 (5)
C8—N3—H3	108.5 (11)	O2—S1—O3	109.98 (5)
C10—N3—H3	108.3 (11)	O3—S1—S2	107.47 (4)
C10—N3—C8	109.88 (9)	H4C—O4—H4D	103.3 (18)
C12—N3—H3	110.8 (11)	H5C—O5—H5D	100.6 (18)
			
N1—C1—C2—N2	10.05 (13)	N4—C7—C8—N3	−13.10 (12)
N1—C3—C4—N2	10.16 (13)	N4—C9—C10—N3	−13.22 (13)
N1—C5—C6—N2	11.29 (14)	N4—C11—C12—N3	−14.10 (12)
C1—N1—C3—C4	55.01 (12)	C7—N4—C9—C10	−51.09 (12)
C1—N1—C5—C6	−66.86 (12)	C7—N4—C11—C12	67.80 (11)
C2—N2—C4—C3	−65.61 (12)	C8—N3—C10—C9	67.72 (11)
C2—N2—C6—C5	52.04 (13)	C8—N3—C12—C11	−52.05 (11)
C3—N1—C1—C2	−66.56 (11)	C9—N4—C7—C8	66.64 (11)
C3—N1—C5—C6	53.66 (12)	C9—N4—C11—C12	−50.30 (12)
C4—N2—C2—C1	53.54 (12)	C10—N3—C8—C7	−52.78 (11)
C4—N2—C6—C5	−66.11 (13)	C10—N3—C12—C11	68.63 (11)
C5—N1—C1—C2	54.47 (12)	C11—N4—C7—C8	−51.34 (11)
C5—N1—C3—C4	−65.72 (12)	C11—N4—C9—C10	66.62 (12)
C6—N2—C2—C1	−65.02 (12)	C12—N3—C8—C7	67.68 (11)
C6—N2—C4—C3	53.35 (12)	C12—N3—C10—C9	−52.85 (11)

### Hydrogen-bond geometry (Å, º)

**Table:**

*D*—H···*A*	*D*—H	H···*A*	*D*···*A*	*D*—H···*A*
N1—H1···O1	0.887 (17)	1.861 (17)	2.7380 (12)	169.6 (15)
N3—H3···O3	0.850 (18)	2.030 (17)	2.8003 (12)	150.4 (15)
O4—H4*C*···O2^i^	0.79 (2)	2.02 (2)	2.8121 (13)	173.4 (18)
O4—H4*D*···O3	0.81 (2)	2.04 (2)	2.8511 (13)	175.1 (18)
O5—H5*C*···N4^ii^	0.85 (2)	2.10 (2)	2.9273 (13)	163.9 (19)
O5—H5*D*···O4	0.91 (2)	1.94 (2)	2.8449 (14)	173.2 (19)

Symmetry codes: (i) *x*−1, *y*, *z*; (ii) −*x*, −*y*+1, −*z*+1.
